# Impact of outdoor air pollution on the incidence of pertussis in China: a time-series study

**DOI:** 10.1186/s12889-023-16530-w

**Published:** 2023-11-13

**Authors:** Yameng Xu, Yizhe Luo, Na Yue, Danyue Nie, Lele Ai, Changqiang Zhu, Heng Lv, Gang Wang, Dan Hu, Yifan Wu, Jiaojiao Qian, Changzhe Li, Jiahong Wu, Weilong Tan

**Affiliations:** 1 Department of Epidemiology, School of Public Health, Nanjing Medical University, Nanjing, 211166 China; 2 Nanjing Bioengineering (Gene) Technology Center for Medicines, Nanjing, China; 3 Hangzhou International Travel Healthcare Center, Hangzhou, 310061 P.R. China; 4 School of Public Heath, Guizhou Medical University, Guiyang, Guizhou 550025 P.R. China; 5 School of Public Health, Nanjing Medical University, 101, Longmian Avenue, Nanjing, 211166 P.R. China

**Keywords:** Air pollution, Distributed lag non-linear model, Pertussis, Risk, Resurgence, Time-series

## Abstract

**Background:**

The increasing number of pertussis cases worldwide over the past two decades has challenged healthcare workers, and the role of environmental factors and climate change cannot be ignored. The incidence of pertussis has increased dramatically in mainland China since 2015, developing into a serious public health problem. The association of meteorological factors on pertussis has attracted attention, but few studies have examined the impact of air pollutants on this respiratory disease.

**Methods:**

In this study, we analyzed the relationship between outdoor air pollution and the pertussis incidence. The study period was from January 2013 to December 2018, and monthly air pollutant data and the monthly incidence of patients in 31 provinces of China were collected. Distributed lag nonlinear model (DLNM) analysis was used to estimate the associations between six air pollutants and monthly pertussis incidence in China.

**Results:**

We found a correlation between elevated pertussis incidence and short-term high monthly CO_2_ and O_3_ exposure, with a 10 μg/m^3^ increase in NO_2_ and O_3_ being significantly associated with increased pertussis incidence, with RR values of 1.78 (95% CI: 1.29-2.46) and 1.51 (95% CI: 1.16-1.97) at a lag of 0 months, respectively. Moreover, PM_2.5_ and SO_2_ also played key roles in the risk of pertussis surged. These associations remain significant after adjusting for long-term trend, seasonality and collinearity.

**Conclusions:**

Overall, these data reinforce the evidence of a link between incidence and climate identified in regional and local studies. These findings also further support the hypothesis that air pollution is responsible for the global resurgence of pertussis. Based on this we suggest that public health workers should be encouraged to consider the risks of the environment when focusing on pertussis prevention and control.

**Supplementary Information:**

The online version contains supplementary material available at 10.1186/s12889-023-16530-w.

## Introduction

Pertussis has been resurging worldwide in the past two decades. Pertussis epidemics have been reported increasingly in East Asia (ie, mainland China, India), Europe (ie, United Kingdom), North America (ie, America and Canada), and Oceania (ie, Australia) since 2008 [1–4]. One study indicated that in 2012, the rate of pertussis in the U.S. was higher than in any previous year since the vaccine was first implemented [2]. A burst of pertussis incidence also appeared in China in recent years and has lasted till today.

Pertussis is a highly contagious respiratory disease caused by the bacterium Bordetella pertussis [5]. After an incubation period of about 1 to 3 weeks (usually 7 to 10 days), patients develop apparent symptoms, from a runny dry cough to a progressive spasmodic cough, the latter lasting for up to 2 months [6]. The pathophysiologic mechanism involves inflammation and spasm of the respiratory tract. The pathogen adheres to the respiratory epithelium and releases toxins causing an inflammatory response that leads to swelling of the respiratory mucosa, increased secretions and decreased mucus membrane secretion. This eventually causes narrowing and obstruction of the respiratory tract, shortness of breath, and coughing. Respiratory muscle spasm will exacerbate this phenomenon and prolong the onset [7–9].

The epidemiology of pertussis appears to be the result of a combination of factors such as un-vaccination, reduced vaccine efficacy, gene mutation in Bordetella pertussis, environmental factor, and climate change [10, 11]. Air pollution has been recognized as a pervasive public health threat [12]. Previous studies have reported the relationship between weather parameters and pertussis [6, 13, 14]. Through regression tree models and DLNMs, Zhang et al. revealed that temperature change, high temperature, and relative humidity increased pertussis risk, temperature found to be a key driver of pertussis infection. Interestingly, the association between pertussis infection and meteorological factors was stronger in people over 10 years old [14]. By studying the relationship between meteorological parameters and the risk of pertussis in Chongqing, China from 2004 to 2018, Wang et al., have shown that for each unit increase in monthly average temperature, the number of monthly pertussis cases increased by 19.496% (95%CI 2.368-39.490%), and meteorological factors such as air pressure and precipitation were also linked to pertussis [6]. However, the small size of the target population, or the assessment of the effect of a single meteorological parameter on pertussis in these studies, was not sufficient to compare the changes of disease incidence and show the long-term role of climate change in the spread of pertussis. What is more, very few studies have explored the impact of air pollutants on this respiratory disease. Therefore, it is necessary to conduct a study that also considered common air pollutants such as (PM_2.5_, PM_10_, SO_2_, CO, NO_2_, and O_3_), to comprehensively elucidate the long-term quantitative association between atmospheric pollutants and pertussis incidence to address the growing threat posed by pertussis to public health.

In this observational study, we aimed to assess changes in pertussis epidemiology, using DLNM models, to explain the complex causes of pertussis surges and the associations between six air pollutants and pertussis, and discuss how to manage these factors to prevent future epidemics and provide early warning information and reference for pertussis epidemic.

## Materials and methods

### Data collection

**Fig. 1:**
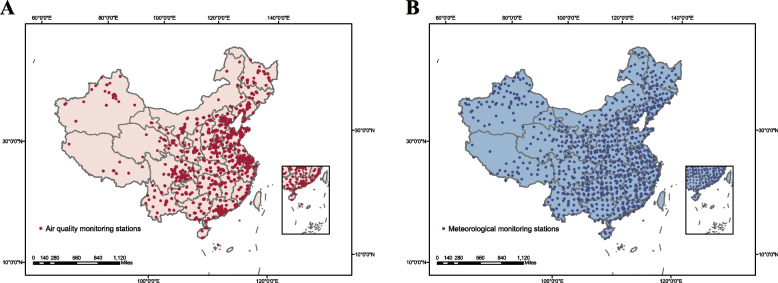
Geographical location of air quality monitoring stations (during 2013-2018) and meteorological monitoring stations (2004-2018) in China. Air quality monitoring stations (*N=*393); meteorological monitoring stations (*N=*839)

We obtained pertussis cases data from the China Public Health Science Data Center (https://www.phsciencedata.cn/Share/en/index.jsp.). Population data of the corresponding periods were obtained from the National Bureau of Statistics of the People’s Republic of China (http://data.stats.gov.cn). All notified cases were diagnosed according to detailed instructions issued by the National Health Commission in 2007 (http://www.nhc.gov.cn/wjw/s9491/201410/52040bc16d3b4eecae56ec28b3358666.shtml). The monthly average air pollutant concentrations (PM_2.5_, PM_10_, SO_2_, CO, NO_2_, and O_3_) and meteorological data (sunshine, mean wind speed, precipitation, relative humidity, and mean temperature) of 31 provinces in China from 2013 to 2018 were obtained from 393 National Air Quality Monitoring Stations (https://www.epmap.org/) and 839 meteorological monitoring stations (https://data.cma.cn/), respectively (Fig. 1).

### Statistical analysis

We analyzed the association between monthly trends in atmospheric pollutants and monthly incidence of pertussis. Since meteorological conditions can affect the concentration of air pollutants, we incorporated five common meteorological conditions into the DLNM model to improve the accuracy of the results. The effects of air pollutants on pertussis were evaluated using DLNM models.

DLNM were based on traditional model ideas such as generalized additive models and distributed lag linear models [15]. The main idea was to add a lag dimension to the exposure-response relationship through a cross basis function. In this way, the change distribution of its effect in the independent variable dimension and the lag dimension was described at the same time.

Both single-pollutant and multi-pollutant models were used to analyze the effect of air pollutant variables on pertussis incidence. The single-pollutant model considered not only air pollutants, but also long-term trend, meteorological conditions, quartiles of average incidence, and incidence in the previous month. The final model looked like this:

$$\mathrm{Model}\;1:\;\mathrm{Log}\;\lbrack\mathrm E(\mathrm{Yt})\rbrack\;=\mathrm\beta\;+\;\mathrm{cb}\;(\mathrm{Kt},\;2,\;\mathrm\beta1)+\mathrm S1\;(\mathrm x)\;+\;\mathrm S2\;(\mathrm z)\;+\;\mathrm S3\;(\mathrm m)\;+\;\mathrm S5\;(\mathrm{Region})\;+\;\mathrm S6\;(\mathrm{Season})$$

Where t is the observation month; [E(Yt)] is the observed pertussis cases in year Yt, and β is the intercept of the whole model; cb (Kt, 2, and β1) is the cross basis of K, and β1 is an estimate of the effect of K in a given lag month t, with the maximum delay month set to 2; K is one of the four air pollutants. S1-3 are "average sunlight", "average temperature", and "average wind speed" representing meteorological factors associated with pertussis incidence; "Region" and "Season" represents the covariates; month is the ordinal variable of January in a year; and S() is the penalized spline function. In this study, we used cubic spline functions S1-3 to adjust for confounders in the model, and S5 (Region) and S6 (Season) to adjust for monthly confounders.

The multi-pollutant model took air pollutants and meteorological conditions (sunshine, wind speed, and mean temperature) associated with pertussis incidence into account, other adjustment variables in the model were long-term trend, seasonal trend, quartile arrays of mean incidence, and the incidence in the previous month. The final model looked like this:

$$\mathrm{Model}:\;\mathrm{Log}\;\lbrack\mathrm E(\mathrm{Yt})\rbrack=\beta+\mathrm{cb}(\mathrm{Kt},\;2,\;\beta1)+\mathrm S1(\mathrm x)+\mathrm S2(\mathrm z)+\mathrm S3(\mathrm m)+\mathrm S5(\mathrm{Region})+\mathrm S6(\mathrm{Season})+\mathrm S7(\mathrm{lag}.\mathrm{value}1)$$

where t is the observation month; [E(Yt)] is the observed pertussis cases in year Yt, and β is the intercept of the whole model; cb (Kt, 2, and β1) is the cross basis of K, and β1 is the estimated impact of K at a specific lag month t, with the maximum delay month set to 2; K is the sum of the four air pollutants. S1-3 are "average sunshine," "average temperature," and "average wind speed," representing meteorological factors associated with pertussis incidence; "region", "season" and "lag.value1" covariates; "lag.value1" represents the average incidence in the previous month; and S() is a penalized spline function. In this study, we used cubic spline functions S1-3 to adjust for confounders in the model, and S5 (Region) and s6 (Season) to adjust for monthly confounders.

The construction of the basis function used the crossbasis () function in the “dlnm” package. The exposure-response dimension fitting was performed using natural cubic splines with node positions set at the 10_th_, 75_th_ and 90_th_ percentiles. The exposure-lag dimension fitting also used the natural cubic spline function, the lag period was selected to be seven months, and the node positions were set using the logarithmic equal spacing method provided by the package “dlnm” (version 4.1.3, https://cran.r-project.org/web/package/dlnm/index.html) [16]. Second, according to the AIC+BIC criterion we selected the degree of freedom, and chose the degree of freedom with the smallest AIC+BIC value to build the final model.

## Results

### Descriptive analysis

From 2013 to 2018, 79 757 cases were reported in China. On average, the monthly mean concentration of PM_2.5_, PM_10_, SO_2_, NO_2_, O_3_ and CO were 50.65 μg/m^3^, 87.67 μg/m^3^, 24.5 μg/m^3^, 33.19 μg/m^3^, 84.36 μg/m^3^, and 0.98 mg/m^3^, respectively (Table 1).

**Table 1:** Descriptive statistics for monthly pertussis cases and air pollution concentrations in China, 2013-2018

**Variables**	**Mean**	**SD**	**Min.**	**P25**	**P50**	**P75**	**Max.**	**China Class II Limit**
No. of cases	692	689	93	276	494	869	3635	/
PM_2.5_ (μg/m^3^)	50.65	28.91	6.29	30.72	43.98	63.36	216.84	35
PM_10_ (μg/m^3^)	87.67	42.99	16.57	56.8	77.52	110.04	319.58	70
SO_2_ (μg/m^3^)	24.5	21.71	2.2	11.57	17.85	28.86	195.29	60
NO_2_ (μg/m^3^)	33.19	13.19	7.86	23.45	31.21	41	94.55	40
O_3_ (μg/m^3^)	84.36	31.08	12.06	60.86	81.98	106.5	184	160
CO (mg/m^3^)	0.98	0.43	0	0.74	0.92	1.11	4.29	4

/: Not available, *SD* Standard deviation, *min* minimum, *P25 25*_*th*_ Percentile, *P50* Median, *P75 75*_*th*_ Percentile, *max* maximum, *PM*_*2.5*_ Particulate matter of <2.5 μm, *PM*_*10*_ Particulate matter of <10 μm, *SO*_*2*_ Sulfur dioxide, *NO*_*2*_ Nitrogen dioxide, *O*_*3*_ Ozone, *CO* Carbon monoxide

### DLNM model

**Fig. 2:**
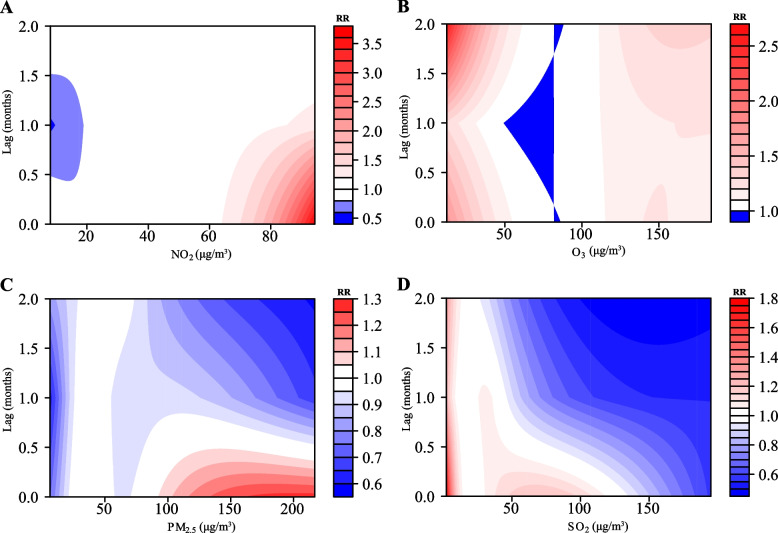
Contour plots of exposure-response relationships between pertussis incidence and four air pollutants in the single-pollutant model. **A**-**D** Four air pollutants associated with (PM_2.5_, SO_2_, NO_2_ and O_3_) pertussis incidence from 2013 to 2018. The reference level was set to the median value of the corresponding variable. The Y-axis represents the lag period from 0 to 2 months. The e-axis represents the range of observations for each variable. RR stands for relative risk, red stands for RR>1, white stands for RR=1, and blue stands for RR<1

**Fig. 3:**
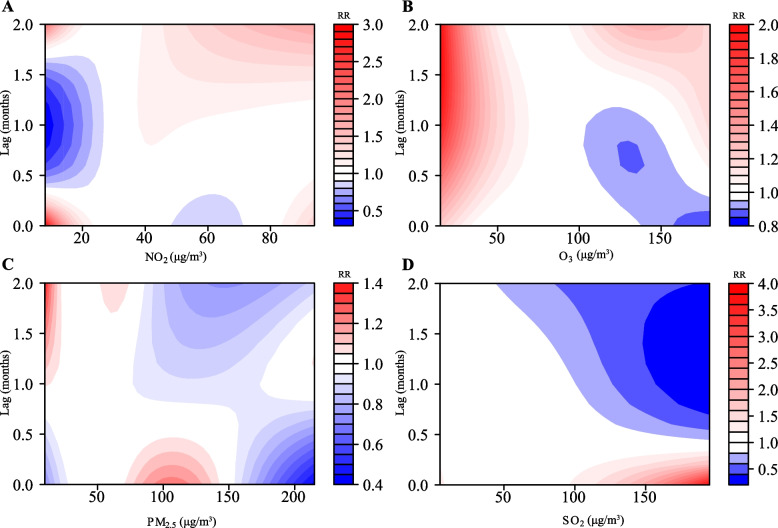
Contour plots of exposure-response relationships between pertussis incidence and four air pollutants in the multi-pollutant model. **A**-**D** Four air pollutants associated with (NO_2_, O_3_, PM_2.5_ and SO_2_) pertussis incidence from 2013 to 2018. The reference level was set to the median value of the corresponding variable. The Y-axis represents the lag period from 0 to 2 months. The X-axis represents the range of observations for each variable. RR stands for relative risk, red stands for RR>1, white stands for RR=1, and blue stands for RR<1

Single-pollutant models showed that four air pollutants (PM_2.5_, SO_2_, NO_2_, and O_3_) were associated with pertussis incidence from 2013 to 2018 (Fig. 2), with relative risks ranging from 0.59-1.27 for PM_2.5_, 0.46-1.79 for SO_2_, 0.57-3.72 for NO_2_, and 0.98-2.61 for O_3_. In the multi-pollutant models (Fig. 3), the relative risk of PM_2.5_ was 0.43-1.38, SO_2_ was 0.21-3.82, NO_2_ was 0.33-2.97, and O_3_ was 0.85-1.99. The maximum RR for PM_2.5_ was 1.38 with exposure to 10 μg/m^3^ PM_2.5_ at lag 2 months. The maximum RR for SO_2_ was 3.82 with exposure to 195μg/m^3^ SO_2_ at lag 1 months. The maximum RR for NO_2_ was 2.97 with exposure to 8 μg/m^3^ NO_2_ at lag 0 months. The maximum RR for O_3_ was 1.99 with exposure to 15 μg/m^3^ O_3_ at lag 1.6 months.

### The lag relationship between air pollutants and the incidence of pertussis

The results of DLNMs were shown in DLNMs, we used median PM_2.5_, median SO_2_, median NO_2_, and median O_3_ as references, and then RR of pertussis incidence with lag 0-2 months was calculated with the extremely low (2.5_th_), 25_th_, 75_th_ and extremely high (97.5_th_) percentile of PM_2.5_, SO_2_, NO_2_, and O_3_, respectively.

**Fig. 4:**
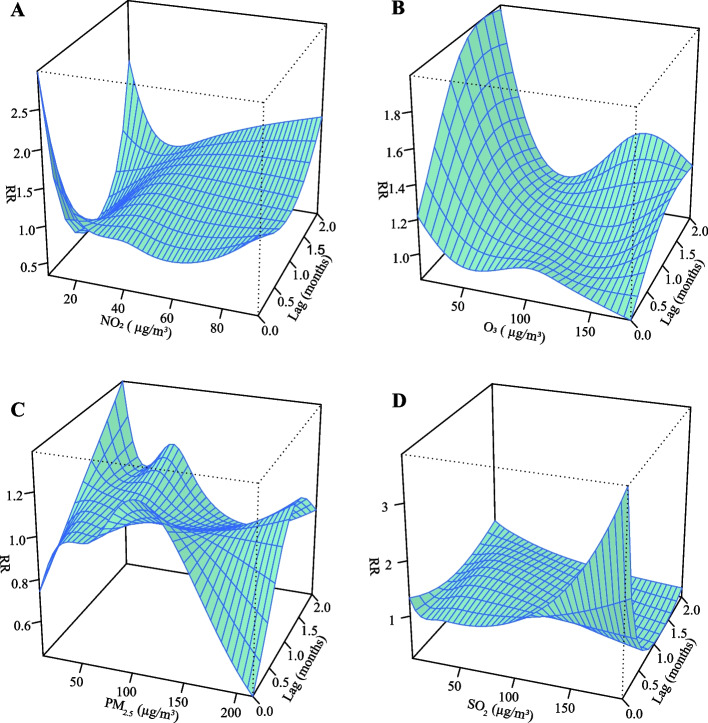
3-D plot of relative risk of pertussis onset (RR) versus lag time for exposure to four air pollutants. **A**-**D** Four air pollutants associated with (NO_2_, O_3_, PM_2.5_ and SO_2_) pertussis incidence from 2013 to 2018. The X-axis represents the exposure concentration of the four pollutants,the Y-axis represents the lag period from 0 to 2 months, the Z-axis represents the relative risk of each variable (NO_2_, O_3_, PM_2.5_ and SO_2_)

**Fig. 5:**
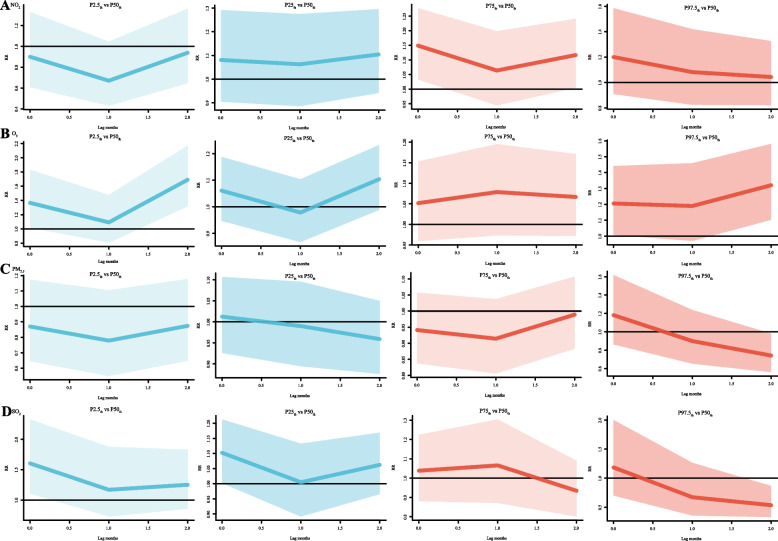
Summary of relative risk curves for different lags between air pollutants and pertussis incidence in single-pollutant models from 2013 to 2018. The Y-axis represents the relative risk of each variable (NO_2_, O_3_, PM_2.5_ and SO_2_), the X-axis represents the lag period from 0 to 2 months. Lines represent means estimated using the DLNM single-pollutant model, shaded areas represent 95% confidence intervals

The 3-D plots for multiple pollutants show that the RR values would increase when the NO_2_ and SO_2_ concentrations increase at a lag of 1 and 0 months (Fig. 4). Furthermore, single-pollutant plots presented in Fig. 5 showed that under the 75_th_ quantile of NO_2_, RR for lag 0 months (RR = 1.15, 95% CI: 1.03-1.28) and RR for 2 months (RR = 1.12, 95% CI: 1.01-1.24) were significantly high. However, under extremely low (2.5_th_) ,25_th_ and high (97.5_th_) NO_2_, no statistical significance in RR was observed; Under the 2.5_th_ quantile of O_3_, the RR for lag 0 months (RR = 1.37, 95% CI: 1.02-1.83) and the RR for lag 2 months (RR = 1.69, 95% CI: 1.32-2.17) were significantly high. In addition, Under the 97.5_th_ quantile of O_3_, the RR for lag 0 months (RR = 1.21, 95% CI: 1.00-1.44) and the RR for lag 2 months (RR = 1.32, 95% CI: 1.10-1.58) were significantly high. And under the 25_th_ and 75_th_ percentile of O_3_, no statistical significance in RR was observed. Under the 2.5_th_, 25_th_, and 75_th_ percentile of PM_2.5_, no statistical significance in RR was observed; under the 2.5_th_, 25_th_ quantile of SO_2_, RR for lag 0 months (RR = 1.60, 95% CI: 1.10-2.33), RR for lag 0 months (RR = 1.10, 95% CI: 1.00-1.21), were significantly high, respectively. Under the 75_th_ percentile of SO_2_, no statistical significance in RR was observed.

**Fig. 6:**
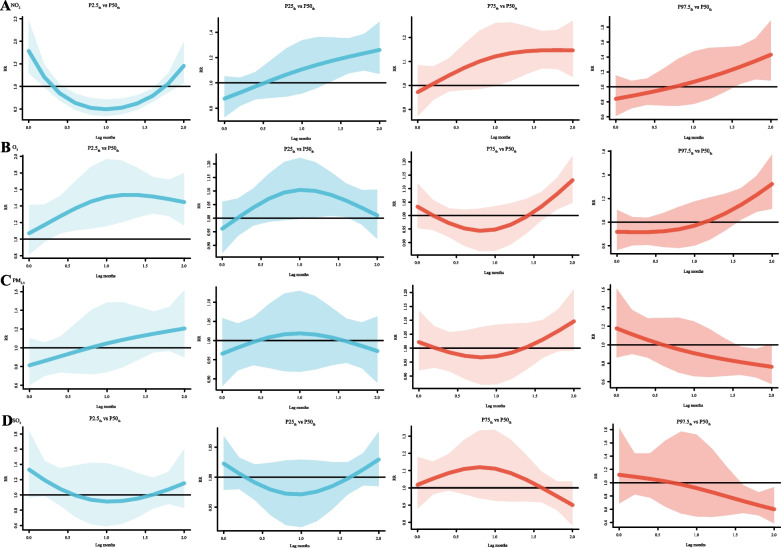
Summary of relative risk curves for different lags between air pollutants and pertussis incidence in multi-pollutant models from 2013 to 2018. The Y-axis represents the relative risk of each variable (NO_2_, O_3_, PM_2.5_ and SO_2_), the X-axis represents the lag period from 0 to 2 months. Lines represent means estimated using the DLNM multi-pollutant model, shaded areas represent 95% confidence intervals

Multi-pollutant plots presented in Fig. 6 showed at the 2.5_th_, 25_th_, 75_th_ and 97.5_th_ percentile of NO_2_, the RR for lag 2 months (RR = 1.45, 95% CI: 1.07-1.98), RR for lag 2 months (RR = 1.26, 95% CI: 1.07-1.48), RR for lag 2 months (RR = 1.15, 95% CI: 1.04-1.27), and RR for 2 months (RR = 1.43, 95% CI: 1.09-1.89) were significantly high, respectively. In addition, under extremely low (2.5_th_) NO_2_, the risk was greatest at a lag of 0 months (RR = 1.78, 95% CI: 1.29-2.46). Under the 2.5_th_, 75_th_ and 97.5_th_ quantile of O_3_, the RR for lag 2 months (RR = 1.45, 95% CI: 1.17-1.80), RR for lag 2 months (RR = 1.13, 95% CI: 1.05-1.22) and the RR for lag 2 months (RR = 1.32, 95% CI: 1.22-1.57) were significantly high, respectively. Additionally, under extremely low (2.5_th_) O_3_, the risk was greatest at a lag of 1 months (RR = 1.51, 95% CI: 1.16-1.97). Under the 25_th_ percentile of O_3_, no statistical significance was observed for RR. Lastly, Under the 2.5_th_, 25_th_, 75_th_ and 97.5_th_ percentile of PM_2.5_ and the 2.5_th_, 25_th_ and 75_th_ percentile of SO_2_, no statistical significance was observed for RR.

## Discussion

Our study mainly analyzed the association between monthly air pollution and monthly pertussis incidence in 31 provinces and regions in China from 2013 to 2018. Estimates of the percent change in pertussis cases with a 10-unit increase in air pollutants were based on a large dataset. Through the indicator, the epidemic trend of pertussis can be predicted, targeted prevention and control measures can be taken in advance, and health education and public health interventions for susceptible populations can be carried out.

Over the past 71 years, the pertussis epidemic in China has gone through four stages: high incidence, decline, low incidence, and resurgence. In addition, the incidence of some respiratory infectious diseases has increased over the past decade. Finally, because of the strong link between climate change and respiratory infectious diseases, we believe that climate has played an important role in the resurgence of pertussis.

The research focused on the association between air pollutants and pertussis incidence. A novel discovery of this research was the exposure-response relationship between the cumulative risk of NO_2,_ O_3,_ PM_2.5_ and SO_2_ and the incidence of pertussis. Considering there was no much literature reporting the association between air pollutants and pertussis, we compared the results of our study with other respiratory infectious diseases.

Environmental particulate matter may play an important role in the occurrence and development of respiratory diseases. Our research results revealed that NO_2_ increased the risk of pertussis and this was consistent with recent discovery. One possible mechanism of NO_2_ and other air pollutants was that, regardless of the duration of exposure, high levels of NO_2_ can irritate airways and increase susceptibility to respiratory infections [17, 18]. Compared with adults, children are at a higher risk because they breathe at higher rates and spend more time outdoors and their lungs and immune systems are immature.

Previous studies have shown that exposure to O_3_ in early life can impair lung function and growth [19–21]. In infant rhesus monkeys, sustained exposure to O_3_ caused structural abnormalities in bronchioles and increased susceptibility to antigen-induced airway remodeling [22]. We found significant evidence of spatial heterogeneity in the associations between air pollutants and morbidity across provinces and regions. Many factors may contribute to this variability, including different geographic distributions, long-term air pollution levels, population susceptibility, environmental policy and planning, and different study period lengths.

Our study found that short-term (0-15 lag days) exposure to high concentrations of PM_2.5_ increased the risk of pertussis infection, and some studies reported that short-term exposure to outdoor particulate matter (PM_10_ or PM_2.5_) have been found to reduce lung function and increase hospitalization rates [23–26]. A recent study assessed the association between ambient particulate matter exposure and chronic obstructive pulmonary disease, using logistic regression analyses and multiple linear regression models. The results provided evidence that exposure to higher PM_10_ or PM_2.5_ concentrations increased the risk of developing COPD and decreased the respiratory function [27]. Mortality rates from respiratory diseases have gradually decreased as air pollutant levels have improved [28, 29]. However, studies have shown that even lower levels of ambient air pollutants can have harmful effects on the respiratory system and lungs, suggesting that even at lower concentrations of pollutants, there is still a risk of disease [30].

As a potent lung irritant that can reduce lung function, SO_2_ has been identified as a cause of respiratory symptoms including cough and reduced lung function. Exposure to high levels of SO_2_ can cause severe airway damage. A study examined the relationship between nitrogen dioxide and sulfur dioxide concentrations and children’s lung function in Greece showed that long-term exposure to higher concentrations of air pollutants was associated with subclinical airway narrowing and slower increase of percent forced vital capacity (FVC%) in 8-10-year-old children [31]. Our results also suggest that higher concentrations of SO_2_ increase the risk of pertussis infection during the lag period of 0-30 days.

Over the past 30 years, the Chinese government has implemented policies to improve air quality and ameliorate the adverse effects of air pollutants on public health. Since 2013, air quality has been continuing to improve in most regions. However, this study showed that NO_2_, O_3_, PM_2.5_ and SO_2_ concentrations and their lagged effects were associated with the risk of pertussis, which indicated that high baseline levels of pollution and its impact on pertussis cases would persist.

Using air pollutants and case data from 2013 to 2018, We found a non-negligible association between short-term pollutant exposure and the development of pertussis. Our previous study analyzed the risk of pertussis after long-term (0-15 lag months) exposure to these major air pollutants. However, considering that it has an incubation period of 1-2 weeks, and even the duration of the disease lasts only 4-6 weeks, we adjusted the lag time to 0-2 lag months in the current study. Generally speaking, these findings further support the hypothesis that air pollution is responsible for the global resurgence of pertussis. Finally, we recommend encouraging public health workers to consider NO_2_ and O_3_ risks in response to the pertussis resurgence.

This study has several limitations. First, we could not obtain the data of air pollutants before 2013 because the national air quality monitoring network was only established at 2013. Second, on the basis of only monthly data we were unable to work out the acute effects of air pollutants on pertussis. Third, we were unable to extrapolate the effect of individual exposure levels on pertussis risk from air pollution across provinces. This study focused on the relationship between six air pollutants and pertussis incidence in China from the perspective of time lag effect. We need to further investigated the potential role of air pollutants in accelerating pertussis transmission in the future.

### Supplementary Information

**Additional file 1:** **Fig. S1.** AIC+BIC parameters for selecting the main model.

## Data Availability

All data generated or analysed during this study are included in this published article (and its supplementary information files).

## Abbreviation

DLNM: Distributed lag nonlinear model
