# Supplementary material for: Impact of outdoor air pollution on the incidence of pertussis in China: a time-series study
Source: BMC Public Health. 2023 Nov 13;23:2231. doi: 10.1186/s12889-023-16530-w (PMC10642023; doi:10.1186/s12889-023-16530-w)
Supplement: Supplementary file 1 — Additional file 1: Fig. S1. AIC+BIC parameters for selecting the main model. [file 12889_2023_16530_MOESM1_ESM.docx]

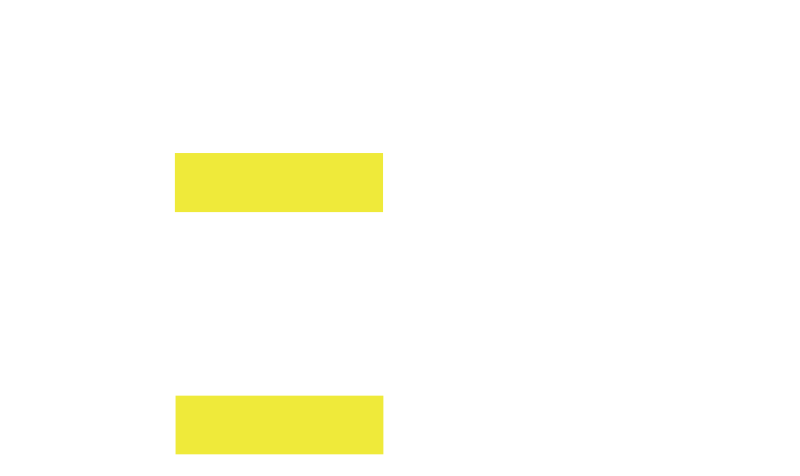


**Fig.S1 AIC+BIC parameters for selecting the main model.**

Yello represents the minimum value of AIC+BIC at different degrees of freedom under this spline curve.

**Model Code**

model <- glm (case~basis.X +ns (seq,6*5) + ns (mean_sunlight,3) + ns (mean_temperature,3) + ns (mean_wind_speed,3) + as.factor (region), family = quasipoisson (), new_ds). “case” represented the number of monthly pertussis cases; “basis.X” represented the cross-basis for air pollutants; “X” represented one of the six air pollutant factors (PM_2.5_, PM_10_, SO_2_, CO, NO_2_, and O_3_); “mean_sunlight”, “mean_temperature”, “mean_wind_speed” represented the meteorological factors associated with pertussis incidence; ns represented nonlinear transformation, which was used to fit nonlinear; “seq” represented the long-term trend; “region” represented the covariate; and “new_ds” represented our dataset.

model1 <- glm (case ~ cb1.no2 + cb1.pm2.5 + cb1.o3 + cb1.so2 +ns (seq,6*5) + ns (mean_sunlight,3) + ns (mean_temperature,3) + ns (mean_wind_speed,3) + factor (region) + factor (season) + lag.value1, family=quasipoisson(), new_ds). “case” represented the number of monthly pertussis cases; “cb1.no2, cb1.pm2.5, cb1.o3, and cb1.so2” represented the cross-basis for air pollutants; “mean_sunlight”, “mean_temperature”, “mean_wind_speed” represented the meteorological factors associated with pertussis incidence; ns represented nonlinear transformation, which was used to fit nonlinear; “seq” represented the long-term trend; “region and season” represented the covariate; “lag.value1” represented mean incidence in the previous month; and “new_ds” represented our dataset.
